# The prevalence and anatomy of accessory navicular bone: a meta-analysis

**DOI:** 10.1007/s00276-024-03459-x

**Published:** 2024-08-13

**Authors:** Kacper Stolarz, Aleksander Osiowski, Maciej Preinl, Maksymilian Osiowski, Barbara Jasiewicz, Dominik Taterra

**Affiliations:** 1 Faculty of Medicine, Jagiellonian University Medical College, św. Anny 12, Krakow, 31-008 Poland; 2 Ortho and Spine Research Group, Zakopane, Poland; 3 Department of Orthopedics and Rehabilitation, Jagiellonian University Medical College, Balzera 15, Zakopane, 34-500 Poland

**Keywords:** Accessory navicular, Os naviculare, Accessory ossicle, Foot and ankle anatomical variant

## Abstract

**Purpose:**

There have been over 40 descriptions of the common developmental variants of the accessory ossicles of the feet. Although predominantly asymptomatic, they sometimes may be linked to painful conditions. One of the most common accessory ossicles in the foot is the accessory navicular bone (AN), located on the medial side of the foot. Our research provides a first meta-analysis on this topic that establishes its frequency by contrasting 39 studies from across the globe.

**Methods:**

Up to February 2024, PubMed and Embase databases were thoroughly searched for research on the AN. Eligible data regarding AN prevalence was extracted. This study strictly adhered to the Preferred Reporting Items for Systematic Reviews and Meta-Analyses (PRISMA) guidelines.

**Results:**

A total of 39 studies, 11,015 patients, and 36,837 feet were analyzed in our study. The pooled prevalence estimate (PPE) of AN was found to be 17.5% (95%CI: 11.5–25.7) and 12.6% (95%CI: 10.1–15.5) in patients and feet analyses, respectively. Accessory navicular occurred bilaterally in 50.0% of patients, with similar distribution in gender-based groups (21.1% of males and 22.0% of females were confirmed with AN). Accessory navicular was most prevalent in the East Asian population (38.4%) and least prevalent in North Americans (8.0%). No significant differences in AN prevalence were found when comparing different imaging modalities (X-ray and cadaver dissection).

**Conclusion:**

Accessory navicular is a common finding in imaging studies. Its prevalence depends on the population covered by the study but is not affected by the patient’s gender or the imaging modality utilized for AN assessment.

**Supplementary Information:**

The online version contains supplementary material available at 10.1007/s00276-024-03459-x.

## Introduction

**Fig. 1:**
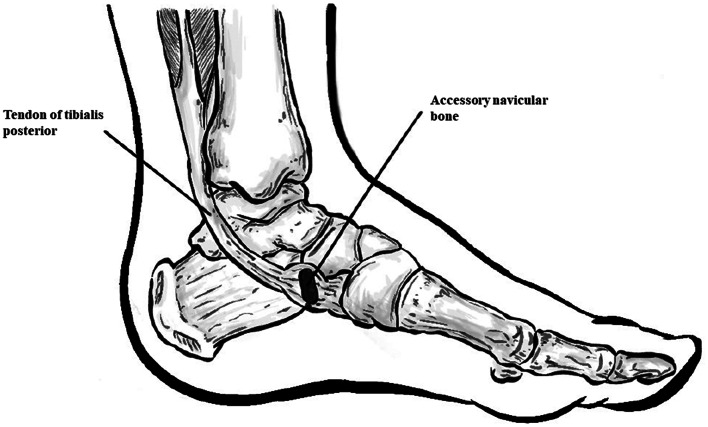
Illustrative diagram presenting the most common location of accessory navicular

The accessory navicular bone, also known as the os tibiale externum or os naviculare secundarium, is an anatomic variant that can be encountered on the medial side of the foot and ankle. Accessory navicular (AN), similarly to other accessory foot ossicles, develops due to the inability of the secondary ossification center to fuse with the primary bone, which in this scenario is the navicular bone [39]. Additionally, the AN may form as a sesamoid bone within the posterior tibial tendon, being, in such a case, anatomically separate from the navicular bone [39] (Fig 1).

**Fig. 2:**
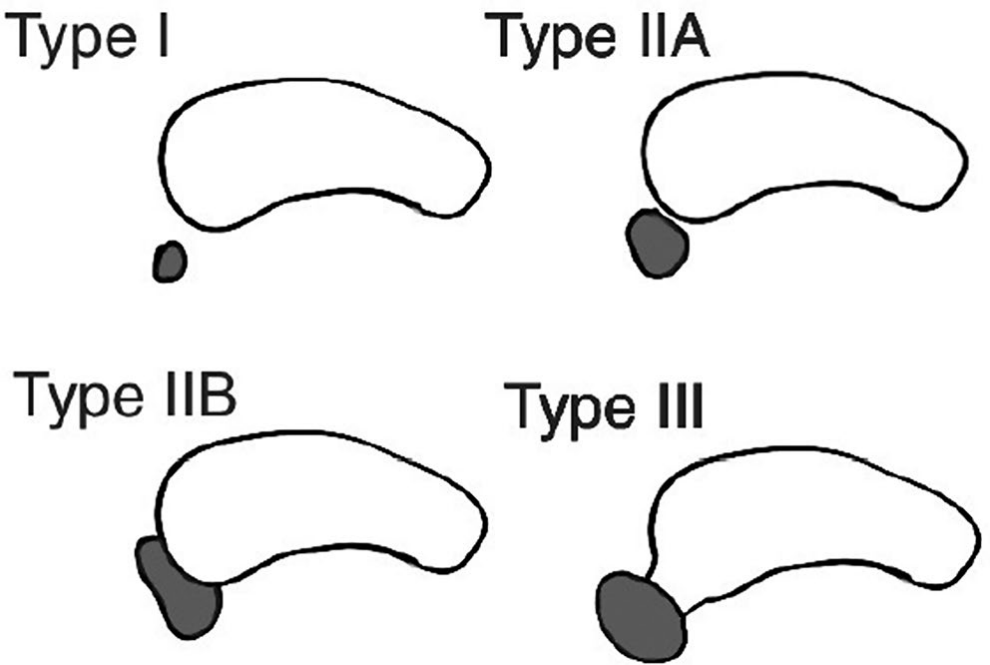
Accessory navicular types distinguished by Coughlin’s classification [13] (light coloured – navicular bone, dark coloured – accessory navicular)

Bauhin, in 1605, is believed to be the first to describe accessory navicular [31]. In 1914, Emil S. Geist proposed the classification that outlines three types of accessory navicular [17]. This classification was later slightly modified by Sella and Lawson in 1987 [60]. Today, the most commonly used AN classification is the one outlined by Coughlin [13] (Fig. 2), which distinguishes the following types of accessory navicular: type I, type II with IIA and IIB subtypes, and type III. A type I accessory navicular is a small, circular, or oval-shaped bone fragment situated within the distal fibers of the posterior tibial tendon (PTT), positioned proximally to the attachment site on the navicular bone [29]. In the literature, type I AN is sometimes referred to as the os tibiale externum [48]. Adjacent to the navicular tuberosity, the type II AN is a bigger osseous core that is triangular or heart-shaped. Its typical size is between 8 and 12 mm. A 1–3 mm synchondrosis made of fibrocartilage and/or hyaline cartilage connects it to the navicular tuberosity, and PTT fibers insert straight onto the accessory ossicle [29, 33, 60, 70]. Type II is divided into two subtypes: type A, which has a smaller acute angle connecting it to the navicular tuberosity, and type B, which is positioned more inferiorly [60]. The type III accessory navicular, also known as the cornuate navicular, is distinguished by a visible yet stable navicular tuberosity, which is assumed to develop from the fusion of an accessory navicular with navicular bone by a bony bridge [29].

The overall prevalence of accessory navicular is believed to fall within the 4–21% range [18, 19, 38, 47, 57], making it one of the most common accessory foot ossicles. When present, accessory navicular is believed to be mostly bilateral (50–90%), with a higher incidence among women [39, 46]. Finding the accessory navicular is usually incidental, as the majority of them remain asymptomatic. However, in small numbers of patients, accessory navicular can be a cause of morbidity [19, 37]. As a result of friction against the footwear, fracture, dislocation or degenerative changes of the AN, patients may struggle with persistent or acute medial foot discomfort or pain, accompanied by swelling, redness, and sensitivity around the navicular tuberosity [14, 39, 48, 67]. It is thought that accessory navicular types I and III are predominantly asymptomatic, while type II accounts for over 70% of all symptomatic cases [10].

The exact prevalence and specific anatomy of accessory navicular remains unclear, as there are many studies presenting contrasting data and no meta-analysis regarding this issue exists to date. Consequently, our study’s objective was to conduct a meta-analysis on the prevalence and anatomy of AN through an evidence-based approach.

## Materials and methods

The Preferred Reporting Items for Systematic Reviews and Meta-Analyses (PRISMA) guidelines were strictly followed by the authors of the presented meta-analysis [51]. Since this is a systematic review with meta-analysis, conducting it required neither participant-informed consent nor ethical approval.

### Search strategy

A comprehensive search of the major scientific electronic databases, including MEDLINE/PubMed and EMBASE, was carried out up to February 2024. No language or date filters were applied by the writers. The MEDLINE/PubMed search query was as follows:

“accessory navicular bone” OR “accessory navicular” OR “os tibiale externum” OR “os naviculare accessorium” OR “os naviculare secundarium OR „os naviculare” OR „naviculare”.

The search techniques for the EMBASE database followed the same general design, taking into account the specific syntactic needs of this search engine. Following the inclusion of the full texts, a manual search was conducted of the featured articles’ reference lists to see if there were any more publications that would be noteworthy and eligible for inclusion.

### Inclusion and exclusion criteria

This meta-analysis included original studies concerning any aspects of accessory navicular prevalence. Exclusion criteria included studies offering inadequate or incomplete data, studies conducted on animals, and studies published in inappropriate format (i.e., meta-analysis, review, case report, and conference report).

For every article, at least two writers carried out separate blind assessments. Studies published in languages other than English were translated by researchers fluent in the appropriate language and included in the final analysis. All the writers had to have an extensive discussion and come to a consensus if there was any disagreement.

### Extraction strategy and outcomes of interest

Data extraction from the included papers was done by two independent authors using a predetermined electronic spreadsheet. The following data was obtained: name of the primary author, year of publication, study design (retrospective/prospective), description of the population (number of participants, country, age, sex), number of identified accessory navicular, accessory navicular subtypes, and imaging modality utilized in the original study (X-ray, CT, MRI, or cadaver dissection). The prevalence of accessory navicular was calculated as a percentage of all patients or feet (according to what was reported by the authors of the original studies) in which accessory navicular was identified. Since not all studies reported data that allowed to recalculate prevalence based only on patients or feet, a consensus was reached that both prevalence rates were reported.

### Quality assessment

The quality and reliability of the included studies were assessed by the reviewers using the AQUA tool [23]. To put it briefly, the tool’s purpose was to look for possible bias [23]. The research looked at five domains: (1) subject and objective; (2) study design; (3) methodology characterization; (4) descriptive anatomy; and (5) reporting of results [23]. Each domain was assigned a risk of bias category, such as “low”, “high”, or “unclear” [23]. The domain was determined to have a “high” risk of bias if a “no” response was given to any signaling question within any of the categories, whereas all “yes” responses indicated a “low” risk of bias [23]. When a study with inconsistent data did not allow for a clear assessment, the “unclear” option was selected [23].

### Statistical analysis and graphic design

The meta-analysis was performed for one outcome: accessory navicular prevalence. The division into subgroups was based on the patients’ gender, country of study’s origin, and imaging modality utilized in the study. Two reviewers performed the statistical analysis. The authors conducted the calculations using Comprehensive Meta-Analysis v4 software [5]. The pooled prevalence estimates (PPE) of accessory navicular were computed using a random effects model. To find meaningful differences between the subgroups that were the subject of the analysis, confidence intervals (CIs) were employed. The absence of a statistically significant difference between the two rates was indicated if their confidence intervals overlapped. χ2 and I2 were used to measure the heterogeneity of the included studies. In relation to the I2 statistic, the heterogeneity was found to “may not be significant” at values of 0–40%, “may indicate moderate heterogeneity” at 30–60%, “may indicate substantial heterogeneity” at 50–90%, and “may represent considerable heterogeneity” at 75–100%. When it comes to interpreting heterogeneity, Cochrane’s Q p value < 0.1 was considered significant. Doi plot with LFK index was utilized to assess potential small-study effect indicating publication bias [16]. LFK index was interpreted as follows: absolute values between 0 and 1 = no significant asymmetry (no significant small-study effect); absolute values between 1 and 2 = minor asymmetry (might suggest small-study effect); absolute values greater than 2 = major asymmetry (strongly suggesting presence of small-study effect). Additionally, MetaXL version 5.3 was used to generate forest and Doi plots while Procreate version 5.3 was utilized to create Figs. 1 and 2.

## Results

### Acquiring the studies

**Fig. 3:**
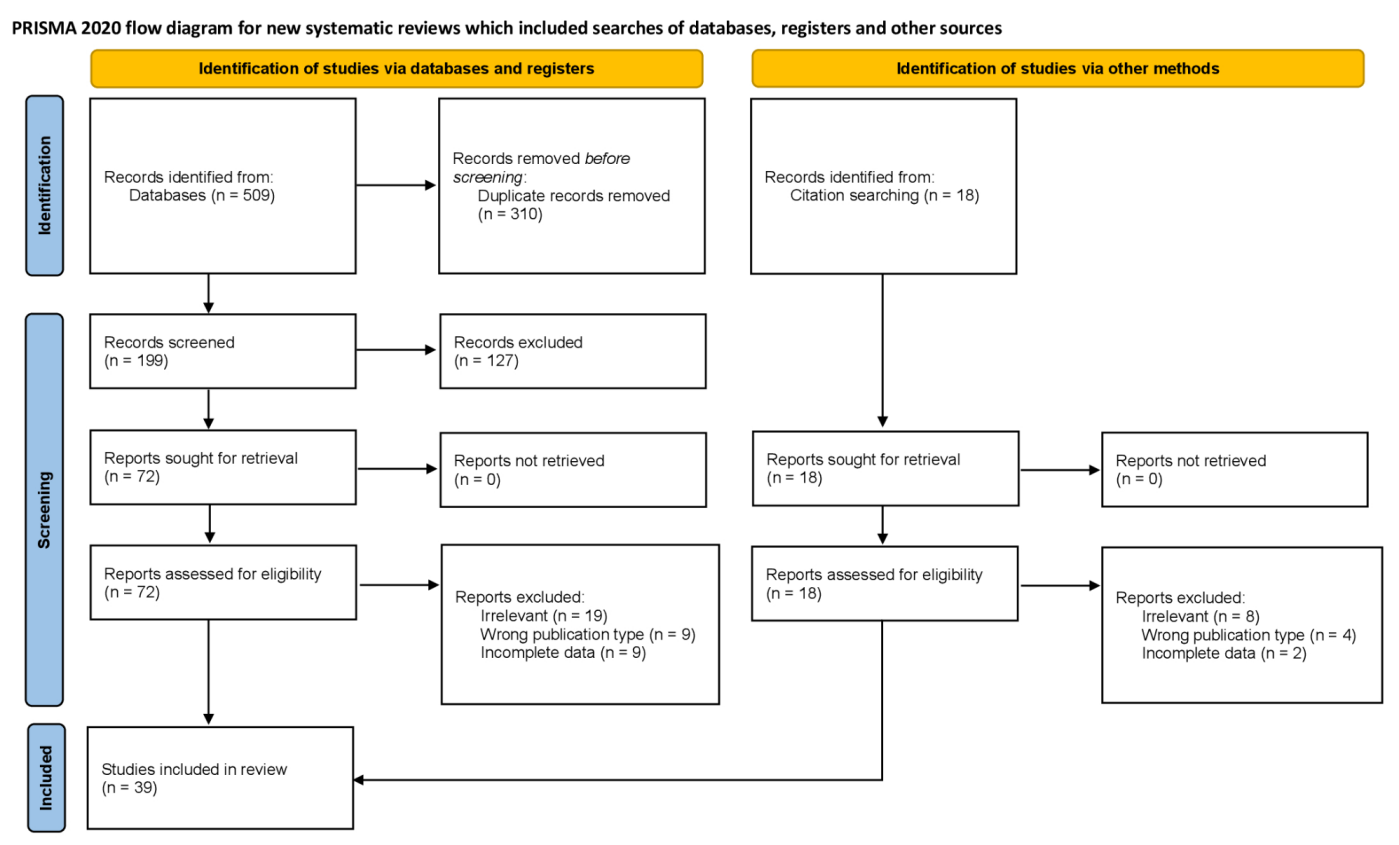
Flowchart of the meta-analysis

A total 199 unique references were found by the database search after duplicates were eliminated. Eighteen more were acquired by the authors from reference search. A total of 72 papers were assessed in full text. This meta-analysis is based on 39 studies that were found after an eligibility evaluation [1, 2, 4, 6–9, 11, 12, 17, 21, 22, 24, 25, 27, 28, 30, 32, 34, 35, 40–43, 45, 48–50, 52–54, 58, 59, 62, 63, 65, 66, 68, 69]. The PRISMA flowchart that describes the inclusion procedure is shown in Fig. 3.

### The characteristics of the included studies

The included studies were published between 1892 and 2022. Sixteen studies reported AN prevalence in a total of 11,015 patients. They were conducted in Turkey (3 studies, 1911 patients), South Korea (2 studies, 1242 patients), Italy (2 studies, 585 patients), Canada (1 study, 3619 patients), China (1 study, 1625 patients), Saudi Arabia (1 study, 503 patients), Singapore (1 study, 439 patients), Germany (1 study, 323 patients), Serbia (1 study, 270 patients), Denmark (1 study, 250 patients), Greece (1 study, 148 patients), and the US (1 study, 100 patients). Thirty-five studies reported AN prevalence on a total of 36,837 feet. They were conducted in Germany (7 studies, 9643 feet), Turkey (5 studies, 5937 feet), Japan (4 studies, 5689 feet), the US (4 studies, 1403 feet), South Korea (3 studies, 3016 feet), Jordan (2 studies, 2240 feet), England (2 studies, 1900 feet), Italy (2 studies, 1124 feet), Singapore (2 studies, 959 feet), China (1 study, 3250 feet), Saudi Arabia (1 study, 1006 feet), Denmark (1 study, 500 feet), and Greece (1 study, 170 feet). The analysis was mainly focused on overall AN prevalence and AN subtypes prevalence, with the secondary end points focused on distribution in subgroups based on gender, geographic origin, and imaging modality utilized to assess the prevalence of AN. Distribution in males and females was reported in 12 studies; 39 studies mentioned the applied imaging modality and provided sufficient data to be included in geographic analysis. Table 1 provides specific details about the studies included in the meta-analysis.

**Table 1:** Detailed characteristics of studies included in the meta-analysis

Study	Region	N of patients	AN in patients	N of feet	AN in feet
Alsager2022 [1]	Middle East	503	117	1006	194
Bagley2022 [2]	East Asia	-	-	81	9
Bizarro1921 [4]	Europe	-	-	100	2
Burman1931 [6]	Europe	-	-	1000	206
Candan2022 [7]	Middle East	-	-	1651	131
Capecchi1964 [8]	Europe	-	-	964	157
Cheong2017 [9]	East Asia	996	408	1992	691
Cilli2005 [11]	Middle East	-	-	464	24
Coskun2008 [12]	Middle East	984	116	1968	148
Coskun2009 [30]	Middle East	650	72	1300	99
Geist1915 [17]	Europe	100	14	200	19
Harris1947 [21]	North America	3619	178	-	-
Heimerzheim1925 [22]	Europe	-	-	1800	21
Holle1938 [24]	Europe	-	-	1000	100
Huang2013 [25]	East Asia	1625	329	3250	391
Kalbouneh2017 [27]	Middle East	-	-	1240	259
Kalbouneh2021 [28]	Middle East	-	-	1000	137
Kim2021 [32]	East Asia	246	125	492	212
Kir2011 [68]	Middle East	277	83	554	129
Kleinberg1917 [34]	North America	-	-	350	12
Knapik2017 [35]	North America	-	-	261	19
Lee2020 [40]	East Asia	-	-	532	181
Leimbach1937 [41]	Europe	-	-	500	18
Longo2013 [42]	Europe	505	34	-	-
Lunghetti1909 [43]	Europe	80	13	160	20
Matsui1964 [45]	East Asia	-	-	213	21
Nikaido1959 [49]	East Asia	-	-	1315	256
Ochs2021 [50]	Europe	-	-	3823	1278
Perdikakis2010 [52]	Europe	148	34	170	34
Pfitzner1892 [53]	Europe	323	43	-	-
Pfitzner1896 [54]	Europe	-	-	752	81
Schönekeß1935 [58]	Europe	-	-	1324	4
Seehausen2016 [59]	North America	-	-	592	73
StacyNg2022 [48]	East Asia	439	202	878	357
Sugano1957 [62]	Europe	-	-	1244	320
Suzuki1957 [63]	East Asia	-	-	701	181
Trolle1948 [65]	Europe	250	19	500	32
Tsuruta1981 [66]	East Asia	-	-	3460	733
Vasilijevic2010 [69]	Europe	270	31	-	-

N = number, AN = accessory navicular

### Quality assessment

Based on the AQUA tool’s evaluation, the majority of the papers included in this meta-analysis showed a “low” risk of bias in each of the five domains. A few studies (mostly those published in the late 1800s and early 1900s) were classified as having an “unclear” risk of bias, primarily because they followed notably different trends of data reporting. Only a small percentage of studies had been categorized as having a “high” risk of bias in every domain (Table 2).

**Table 2:** Quality assessment of the studies included in the meta-analysis performed with the AQUA tool [23]

	Risk of bias				
Study	Objective(s) and study characteristics	Study design	Methodology characterization	Descriptive anatomy	Reporting of results
Alsager2022 [1]	LOW	LOW	LOW	LOW	LOW
Bagley2022 [2]	LOW	LOW	HIGH	LOW	HIGH
Bizarro1921 [4]	UNCLEAR	UNCLEAR	UNCLEAR	UNCLEAR	UNCLEAR
Burman1931 [6]	UNCLEAR	UNCLEAR	UNCLEAR	UNCLEAR	UNCLEAR
Candan2022 [7]	LOW	LOW	LOW	LOW	LOW
Capecchi1964 [8]	HIGH	HIGH	HIGH	HIGH	HIGH
Cheong2017 [9]	LOW	LOW	LOW	LOW	LOW
Cilli2005 [11]	LOW	LOW	LOW	LOW	LOW
Coskun2008 [12]	LOW	LOW	LOW	LOW	LOW
Coskun2009 [30]	LOW	LOW	LOW	LOW	LOW
Geist1915 [17]	UNCLEAR	UNCLEAR	UNCLEAR	UNCLEAR	UNCLEAR
Harris1947 [21]	HIGH	LOW	HIGH	HIGH	LOW
Heimerzheim1925 [22]	UNCLEAR	UNCLEAR	UNCLEAR	UNCLEAR	UNCLEAR
Holle1938 [24]	UNCLEAR	UNCLEAR	UNCLEAR	UNCLEAR	UNCLEAR
Huang2013 [25]	LOW	LOW	HIGH	LOW	LOW
Kalbouneh2017 [27]	LOW	LOW	LOW	LOW	LOW
Kalbouneh2021 [28]	LOW	LOW	LOW	LOW	LOW
Kim2021 [32]	LOW	LOW	LOW	LOW	LOW
Kir2011 [68]	LOW	LOW	LOW	LOW	LOW
Kleinberg1917 [34]	UNCLEAR	UNCLEAR	UNCLEAR	UNCLEAR	UNCLEAR
Knapik2017 [35]	LOW	LOW	LOW	LOW	LOW
Lee2020 [40]	LOW	LOW	LOW	LOW	LOW
Leimbach1937 [41]	UNCLEAR	UNCLEAR	UNCLEAR	UNCLEAR	UNCLEAR
Longo2013 [42]	HIGH	LOW	LOW	LOW	HIGH
Lunghetti1909 [43]	UNCLEAR	UNCLEAR	UNCLEAR	UNCLEAR	UNCLEAR
Matsui1964 [45]	LOW	LOW	LOW	LOW	LOW
Nikaido1959 [49]	LOW	LOW	LOW	LOW	LOW
Ochs2021 [50]	LOW	LOW	LOW	LOW	LOW
Perdikakis2010 [52]	LOW	LOW	LOW	LOW	LOW
Pfitzner1892 [53]	UNCLEAR	UNCLEAR	UNCLEAR	UNCLEAR	UNCLEAR
Pfitzner1896 [54]	UNCLEAR	UNCLEAR	UNCLEAR	UNCLEAR	UNCLEAR
Schönekeß1935 [58]	UNCLEAR	UNCLEAR	UNCLEAR	UNCLEAR	UNCLEAR
Seehausen2016 [59]	LOW	LOW	HIGH	HIGH	HIGH
StacyNg2022 [48]	HIGH	HIGH	LOW	HIGH	HIGH
Sugano1957 [62]	UNCLEAR	UNCLEAR	UNCLEAR	UNCLEAR	UNCLEAR
Suzuki1957 [63]	HIGH	HIGH	LOW	LOW	LOW
Trolle1948 [65]	LOW	LOW	LOW	LOW	LOW
Tsuruta1981 [66]	LOW	LOW	LOW	LOW	LOW
Vasilijevic2010 [69]	LOW	LOW	LOW	LOW	LOW

### General prevalence

The total number of patients included in the analysis was 11,015. Among them, 1812 individuals were confirmed to have AN. The pooled prevalence estimate (PPE) in this group was 17.5% (95% CI: 11.5–25.7%) (Table 3; Fig. 4a). The analysis also independently included 36,839 feet, of which 6549 AN were identified. The PPE for the feet analysis was 12.6% (95% CI: 10.1–15.5%) (Table 3; Fig. 4b). Twelve studies provided sufficient data of 1526 patients regarding AN laterality. Accessory navicular occurred unilaterally (PPE = 50.0% (95% CI = 35.4-64.7%)) and bilaterally (PPE = 50.0% (95% CI = 35.3-64.6%)) with almost equal frequency (Table 3). Four studies reported the prevalence of AN subtypes in a total of 435 patients with AN, while three papers noted the prevalence of AN subtypes in 487 feet with present AN. In patient analysis, type I AN was the most common (PPE = 41.3% (95% CI = 30.8–52.7%)), while types IIa and IIb were the rarest (PPE = 18.1% (95% CI = 12.4–25.6%) and 15.1% (95% CI = 9.9–22.4%), respectively. The feet analysis provided similar results: type I AN was the most common (PPE = 38.9% (95% CI = 24.7-55.2%)) and types IIa and IIb were the rarest (PPE = 17.6% (95% CI = 13.5-22.8%) and 19.9% (95% CI = 13.9–27.7%), respectively) (Table 4). Small-study effect analysis revealed LFK index values of -0.96 (no asymmetry) and − 3.85 (major asymmetry) for patient-based and feet-based groups (**Supplementary material 1**), respectively.

**Table 3:** Meta-analysis presenting general prevalence of accessory navicular, its laterality, and comparison of its prevalence between gender-based groups

Population	Number of patients/feet examined	Prevalence of AN per patients % (95% CI)	Prevalence of AN per feet % (95% CI)	I-squared % (patients/feet)	Cochrane’s Q *p*-value (patients/feet)
General	11,015/36,837	17.5 (11.5–25.7)	12.6 (10.1–15.5)	98.7/98.6	< 0.001/<0.001
Male	2396/4376	21.1 (12.8–32.6)	12.6 (7.3–20.9)	96.7/97.8	< 0.001/<0.001
Female	3228/4452	22.0 (13.6–33.6)	15.2 (8.6–25.4)	97.6/98.3	< 0.001/<0.001
Unilateral AN	1526/-	50.0 (35.4–64.7)	-	96.0/-	< 0.001/-
Bilateral AN	1526/-	50.0 (35.3–64.6)	-	96.0/-	< 0.001/-

AN = accessory navicular

**Fig. 4a:**
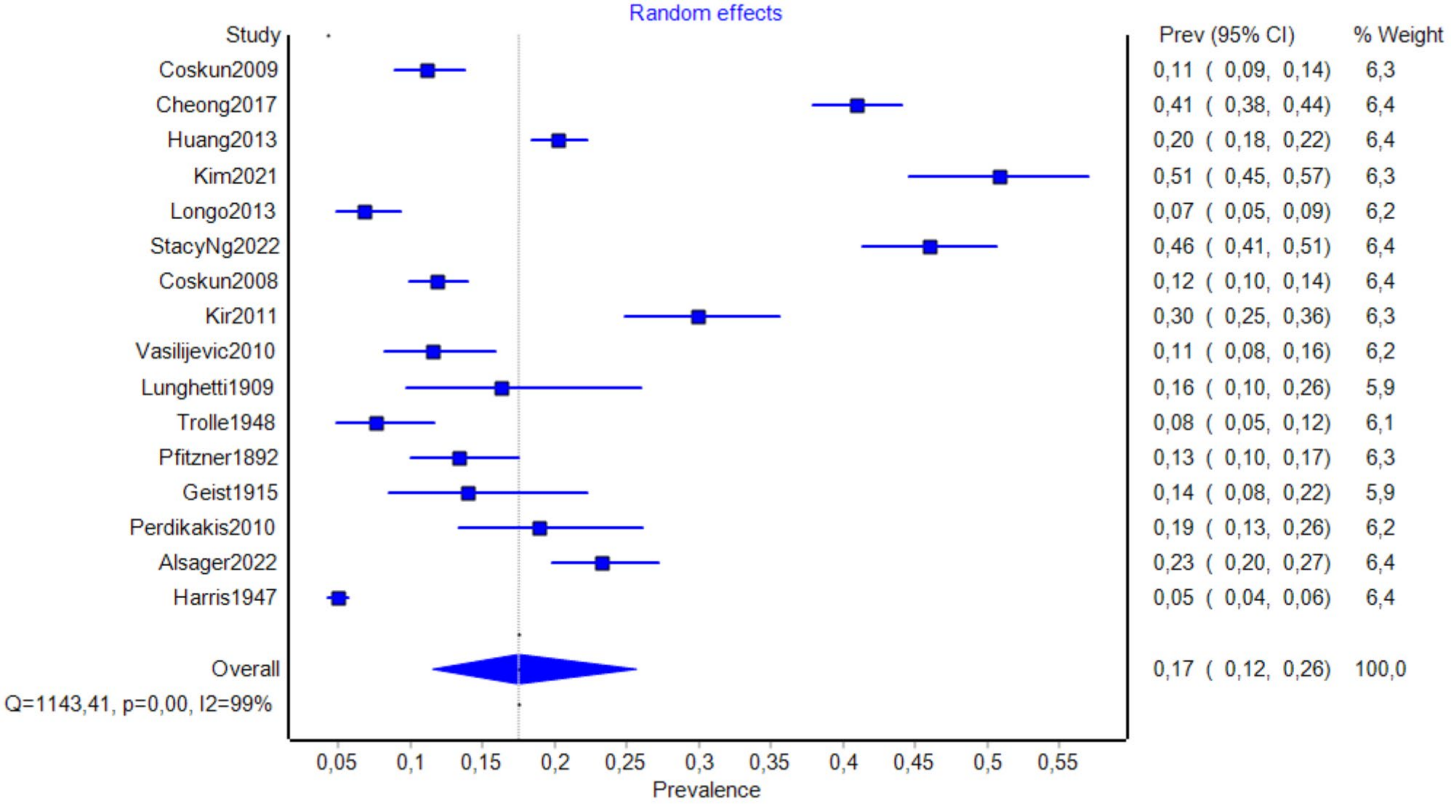
Forest plot presenting general prevalence of accessory navicular (patient analysis)

**Fig. 4b:**
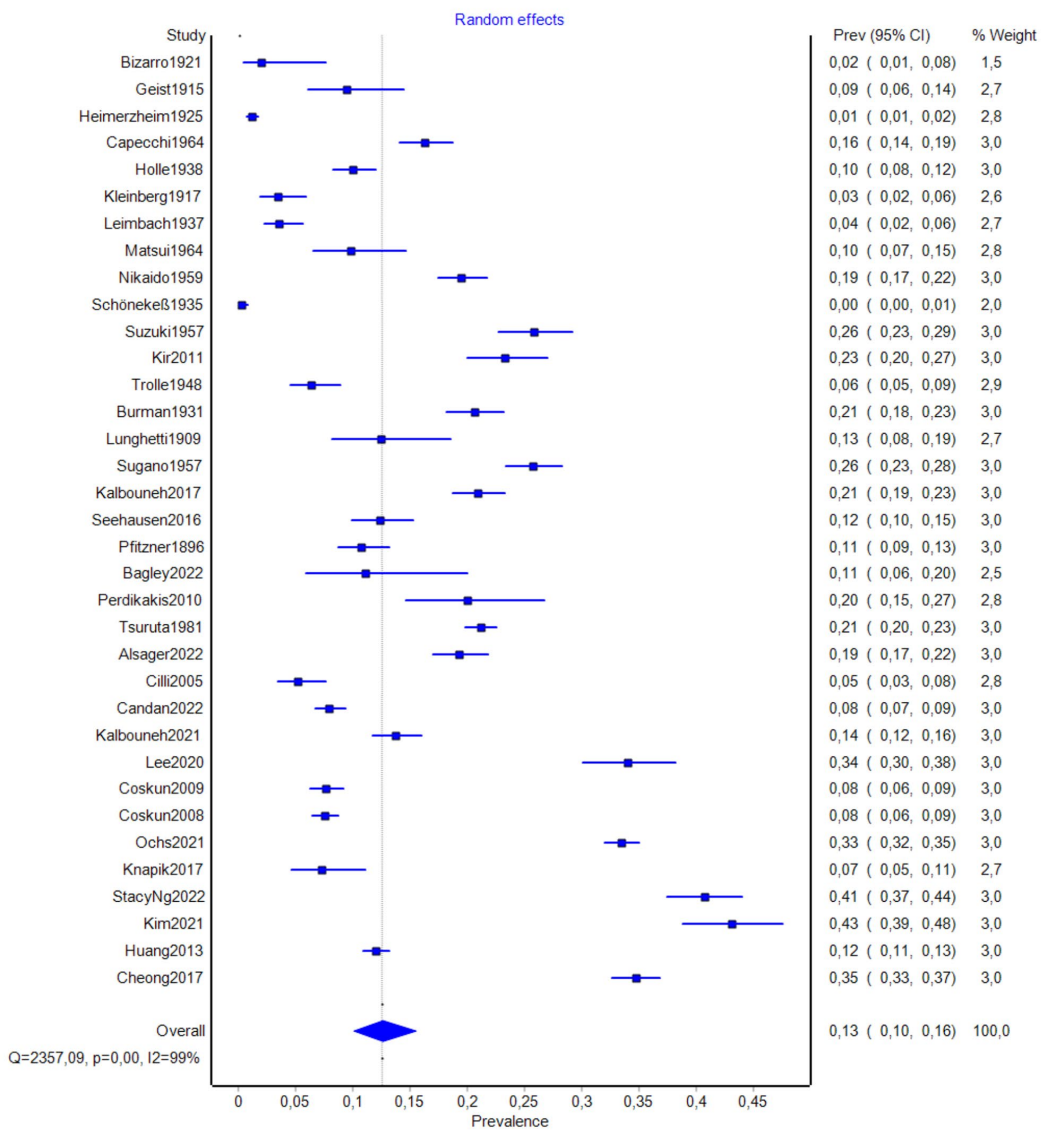
Forest plot presenting general prevalence of accessory navicular bone per feet

**Table 4:** Meta-analysis comparing prevalence of accessory navicular subtypes

ON subtype	Prevalence of AN per patients % (95% CI)	Prevalence of AN per feet % (95% CI)	I-squared % (patients/feet)	Cochrane’s Q *p*-value (patients/feet)
Type I	41.5 (29.7–54.3)	37.8 (27.8–48.9)	68.1/89.7	0.04/<0.001
Type IIa	17.6 (10.9–27.2)	17.7 (11.7–25.8)	44.2/35.0	0.17/0.21
Type IIb	14.6 (8.5–24.1)	19.5 (12.6–28.9)	38.8/57.5	0.19/0.09
Type III	28.2 (18.8–39.9)	27.6 (19.3–37.7)	83.6/32.6	< 0.001/0.22

AN = accessory navicular

### Gender prevalence

Overall, 2396 males and 3228 females were included in the analysis. Accessory navicular was identified in 503 males, resulting in a PPE of 21.1% (95% CI: 12.8–32.6%) in this group. A total of 769 females were reported with the presence of AN. Pooled prevalence estimate in this group was therefore 22.0% (95% CI: 13.6–33.6%). Additionally, a total of 4376 male and 4452 female feet were analyzed. The AN was noted in 738 and 997 feet in men and women, respectively. Pooled prevalence estimate for male feet was 12.6% (95% CI: 7.3–20.9%) and 15.2% (95% CI: 8.6–25.4%) for female feet. The analyses of both patients and feet failed to find a statistically significant difference between gender-based groups (Table 3).

### Geographic origin prevalence

A group consisting of 11,015 patients was found eligible for this analysis. Individuals originated from East Asia (3306 people), the Middle East (2414 people), Europe (1576 people), and North America (3719 people). Pooled prevalence estimate for AN was the highest in East Asia and the lowest in Europe and North America for both patients and feet based analyses. Pooled prevalence estimates for AN in the included populations were as follows: 38.4% (95% CI: 25.2–53.7%) in East Asia, 17.7% (95% CI: 10.4–28.6%) in the Middle East, 12.1% (95% CI: 7.5–18.9%) in Europe, and 8.0% (95% CI: 3.4–17.8%) in North America. Analogous analysis was conducted on feet. A total of 36,837 feet from four world regions (12914 from East Asia, 9183 from the Middle East, 13337 from Europe, and 1403 from North America) were investigated. Pooled prevalence estimates for AN in these populations were as follows: 23.6% (95% CI: 16.7-32.3%) in East Asia, 11.8% (95% CI: 7.6-17.8%) in the Middle East, 8.9% (95% CI: 6.2-12.6%) in Europe, and 7.6% (95% CI: 3.9-14.3%) in North America (Table 5).

**Table 5:** Meta-analysis comparing accessory navicular prevalence between ethnicity-based groups

Region	Number of studies (patients/feet)	Patients’ prevalence % (95% CI)	Feet prevalence % (95% CI)	I-squared % (patients/feet)	Cochrane’s Q *p*-value (patients/feet)
East Asia	4/10	38.4 (25.2–53.7)	23.6 (16.7–32.3)	98.6/98.6	< 0.001/<0.001
Middle East	4/8	17.7 (10.4–28.6)	11.8 (7.6–17.8)	96.2/97.6	< 0.001/<0.001
Europe	6/13	12.1 (7.5–18.9)	8.9 (6.2–12.6)	85.8/98.6	< 0.001/<0.001
North America	2/2	8.0 (3.4–17.8)	7.6 (3.9–14.3)	93.2/85.4	< 0.001/<0.001

### Prevalence based on the type of study

Overall, 10 621 patients were included in the analysis. A total of 9968 patients were examined on the X-rays, while data about the remaining 653 came from the dissection of the cadavers. Accessory navicular was found on 1584 X-rays (PPE = 17.0% (95% CI: 10.3–26.8%), I^2^ = 99.0%, *p* = 0.00) and in 75 cadavers (PPE = 11.9% (95% CI: 4.1–19.5%), I^2^ = 68.6%, *p* = 0.04). A similar analysis was conducted on 36 064 feet. A total of 34 652 feet were examined on X-rays, and 1412 were assessed during cadaver dissections. Accessory navicular was identified in 6161 feet in the X-ray group (PPE = 12.2% (95% CI: 9.6-15.3%), I^2^ = 98.7%, *p* = 0.00), and in 133 feet in the cadaver group (PPE = 9.5% (95% CI: 4.5-19.2%), I^2^ = 76.5%, *p* = 0.01).

## Discussion

The accessory navicular is an accessory ossicle that can be found on the medial side of the foot in close proximity to the medial and inferior margins of the navicular bone and is often embedded in the distal fibers of the posterior tibial tendon (PTT). There is currently a lack of studies systematically analyzing the anatomy and prevalence of AN. Thus, the purpose of our research was to conduct a meta-analysis in order to standardize the data regarding the prevalence of AN with geographical considerations.

Like other accessory foot ossicles, accessory navicular develops as a result of the secondary ossification center’s inability to fuse with the parent bone, the navicular bone. According to Lawson’s study [38], the ossicle formation process takes place between the ages of 8 and 13. However, in the work conducted on human embryos, Trolle [65] observed that numerous accessory ossicles, including accessory navicular, are already preformed in the embryonic stage. This indicates that the ossification and formation of the foot’s accessory ossicles occur at different times, with some of them possibly existing during the prenatal period. Moreover, some authors [8, 25] state that AN may develop due to mechanical stress or with increasing age. Capecchi et al. [8] were able to identify a dependence of AN prevalence on age, with a significant increase in its prevalence at the age of 40. In the study by Huang et al. [25], a similar relation occurred up to the age of 60, with the highest AN prevalence of 29.7% in the 51–60 age group. Accessory navicular predominantly exists as a single accessory bone, however, reports in the literature point out to its correlation with the presence of the os peroneum [50] as well as the possibility of two AN occurring in a single foot [61].

The presence of AN is predominantly asymptomatic, however, a small percentage of patients (≈ 1%) may experience some disturbing symptoms related to its presence [19, 37]. Clinical implications associated with AN include sensitivity, redness, and swelling around the navicular tuberosity, together with chronic or acute medial foot pain or discomfort [14, 39, 48, 67]. Those symptoms might pose a diagnostic dilemma especially in the emergency setting. Accessory navicular can be mistakenly identified as navicular tuberosity avulsion fracture and vice versa. Similar situation has also been reported for other accessory ossicles of the foot [3, 32]. While most can be easily differentiated on radiographs, sometimes more advanced imaging, such as CT, MRI or Tc-99 m bone scan is required [20].

The reason why some patients with AN experience symptoms while others do not is still a matter of debate [36]. Some individuals with AN have been confirmed to have stress fractures, which are often caused by the posterior tibial tendon [29, 39]. When AN is present, the excessive bony tissue forces the PTT to subluxate over the medial malleolus. As a result, the tendon must pull harder to maintain its function, which generates strong tension over the AN, leading to either a stress fracture or lowering the foot arch as the patient experiences a traumatic division of the PTT [29, 39]. While some authors hypothesize that subluxated tibialis posterior tendon stress fractures cause symptoms, others have linked the lowered foot arch and pes planus to an increase in stress on the accessory navicular during weight-bearing exercises [57, 61].

When symptoms related to AN presence occur, a conservative approach is the first line of treatment. For conservative treatment, apart from the use of NSAIDs and ice, arch support is frequently used to elevate the foot arch and reduce pressure from the shoe on the AN, sometimes improving symptoms [37, 39, 47, 55]. When discomfort and pain last for more than a year or two, surgical excision is worth considering [55, 56]. Although the percentage of patients requiring surgical intervention is very low, the removal of the accessory navicular bone usually results in highly favorable surgical outcomes [44, 61]. Previous studies [26] on the simple excision of accessory navicular, reported substantial pain alleviation visible already in the post-operative period, accompanied by a considerable level (76%) of patients’ satisfaction after the follow-up period. An article by Macnicol and Voutsinas [44] examined the outcomes of simple excision of AN and compared them to outcomes in patients after Kidner procedure. When the major navicular bone was shaped to prevent any residual prominence after the accessory navicular was removed a comparable results in alleviating the symptoms were obtained to patients who underwent Kidner procedures [44]. In total, 86% of all patients reported feeling satisfied [44].

To our knowledge, this is the first meta-analysis addressing the problematics of prevalence and anatomy of the accessory navicular. Due to the inconsistency in reporting the AN prevalence in the literature, we decided to undertake this issue by performing the analysis of patient prevalence and foot prevalence separately. The prevalence for patients was 17.5% and 12.6% for feet. Both of those values fit into the range (4–21%) [18, 19, 38, 47, 57] reported previously by numerous authors, yet the difference between them fails to reach statistical significance. The problematics of the difference in patient and foot prevalence, precisely described by Ochs [50], originates from the fact that AN does not always occur bilaterally, and some patients are diagnosed with AN only in one foot. However, considering the substantial variety of the included studies when it comes to reporting data (not every patient had both of his or her feet examined), it was impossible to directly extrapolate the data from patients to feet analysis. Therefore, our analysis of AN bilaterality was based only on 1526 patients and revealed that AN occurred unilaterally and bilaterally with almost equal frequency. This finding does not support the theory that, when present, AN is mostly bilateral [39, 46]. However, neither of our results prove that AN is predominantly unilateral.

The analysis for prevalence of certain AN subtypes (as described by Coughlin [13] in his classification) provided similar results for both patients and feet, with PPEs reaching the highest values for type I AN (41.5% and 37.8% for patients and feet, respectively) and the lowest for type IIb (14.6% for patient analysis) and type IIa (17.7% for foot analysis), with differences being statistically significant for both analyses. This information is worth noticing, as reports in the literature indicate that types IIa and IIb of accessory navicular make up more than 70% of all symptomatic cases, whereas types I and III are primarily asymptomatic [10]. However, the limitation of this observation includes a relatively small sample of patients (435/11,015) and feet (487/36,837) analyzed completely for subtypes prevalence.

Previous studies reported that AN has a higher prevalence among women when compared to the male population [1, 25, 30, 39, 46]. Yet this meta-analysis does not seem to support this hypothesis. Our results showed that AN is present in 21.1% of males and in 22.0% of females. Foot prevalence analysis provided 12.6% and 15.2% PPEs for men and women, respectively. No significant difference between genders was found in the patient nor in the foot prevalence analysis. Surprisingly, only a fraction of authors (8 studies for patients and 6 studies for foot analysis) provided complete data about the gender distribution of AN. However, a total of 5624 patients and 8828 feet underwent the analysis, thus significantly exceeding the samples screened in previous studies covering this matter [1, 25, 30, 39, 46].

Following the study by Stacy NG et al. [48] conducted on the multiethnic population of Singapore, our meta-analysis has also undertaken the problematics of AN prevalence based on geographical origin of the study. Patients’ analysis results revealed that AN prevalence was the highest (38.4%) in East Asia, followed by the Middle East with 17.7% and Europe with 12.1%. The lowest AN prevalence was in North America, where only 8.0% of the patients were diagnosed with AN. Foot prevalence analysis provided similar outcomes: the highest AN prevalence was observed in the East Asian population, while the lowest was in the North Americans. These results compare favorably with the reports from the literature. Stacy NG et al. [48] observed the highest AN prevalence of 50.2% among Chinese individuals and the lowest among Euroasians (20%). The exact mechanism underlying the discrepancy in AN prevalence between populations of different ethnicities remains unknown; however, the genetic factor seems to play a considerable role. Kiter et al. [33] discovered in 2000 that the inheritance of AN is characterized by an autosomal dominant pattern with partial penetrance. Their observation was later confirmed by Dobbs and Walton in 2004 [15]. Eventually, the role of genetics in AN distribution was established by Cheong [9] in 2017 in a Korean family study, which comprised roughly 1000 participants.

We failed to observe a statistically significant relationship between the number of AN and types of studies. Although cadaveric dissection is considered to be a golden standard in anatomy, we were not able to identify the cause of the lowest AN prevalence in cadaver-based studies. The examiner’s proficiency in dissection may affect the accuracy of cadaver dissections. Therefore, AN examined with this method might have been mistakenly omitted, which may have caused lower PPE for dissection when compared to X-ray for both patient and foot prevalence analyses. Moreover, the most common diagnostic, X-ray examination, is only as accurate in determining bone structure as the quality of the images it uses. Because of their great sensitivity and specificity, CT and MRI imaging techniques yield high prevalence of AN as reported previously (50.8% for patient analysis and 43.1% for feet analysis in 1 CT based study [32], 11.1% for feet analysis in 1 MRI based study [2]). However, they were not included in the analysis given the insufficient number of studies performed with these imaging modalities. Therefore, the true prevalence of AN may be underestimated.

The considerable degree of heterogeneity within the included research placed limitations on our investigation. We employed several subgroup analyses to investigate the source of the heterogeneity, but it remained throughout the course of the study. Moreover, a small-study effect analysis revealed major asymmetry in the Doi plot for feet-based prevalence analysis, with an LFK index value of -3.85 strongly suggesting the presence of a small-study effect. This result indicates possible negative publication bias, i.e., studies reporting lower AN prevalence rates may be more likely to be published than those reporting higher prevalence rates in feet-based analysis. This can lead to an underestimation of the actual AN foot prevalence in our meta-analytic findings. Fortunately, no similar effect was found in the patient-based analysis. A further drawback is that the study protocol was not registered before to this systematic review and meta-analysis. The global poll found that while it is advised, this strategy is not frequently employed [64]. The findings’ generalizability may be limited by the lack of research conducted in Australia, Oceania, and South America.

## Conclusions

According to this study, AN is a very common accessory bone with a prevalence of 17.5% overall and no gender differences. The East Asian and Middle East populations have the highest prevalence of AN. In clinical practice, doctors—especially orthopedic surgeons—should be aware that the accessory navicular bone’s prevalence and anatomy. Such knowledge facilitates a more accurate diagnosis when a patient complains of discomfort or conflict in the medial aspect of the foot and ankle.

## Electronic supplementary material

Below is the link to the electronic supplementary material.

Supplementary Material 1

## Data Availability

The original publications provided the data used in the manuscript. Upon request, the corresponding author can supply the entire set of data used in this analysis.
