# Supplementary material for: The prevalence and anatomy of accessory navicular bone: a meta-analysis
Source: Surg Radiol Anat. 2024 Aug 13;46(10):1731–43. doi: 10.1007/s00276-024-03459-x (PMC11405447; doi:10.1007/s00276-024-03459-x)
Supplement: Supplementary file 1 — Supplementary Material 1 [file 276_2024_3459_MOESM1_ESM.pdf]

Supplementary material 1 - Doi plots with LFK index of patient-based evaluation and feet-based evaluation.

The Prevalence and Anatomy of Accessory Navicular Bone: A Meta-Analysis

Surgical and Radiologic Anatomy

Kacper Stolarz, Aleksander Osiowski, Maciej Preinl, Maksymilian Osiowski, Barbara Jasiewicz, Dominik Taterra

Corresponding author:

Dominik Taterra, MD, dominik.taterra@gmail.com, Department of Orthopedics and Rehabilitation, Jagiellonian University Medical College, Zakopane, Poland

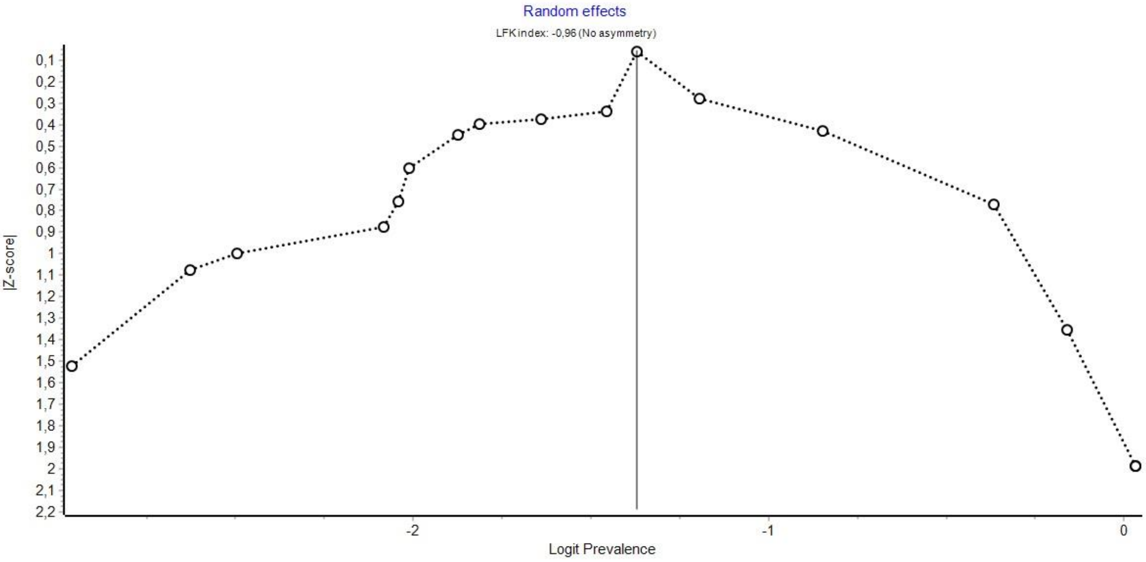

Supplementary figure 1 Doi plot with LFK index of patient-based evaluation

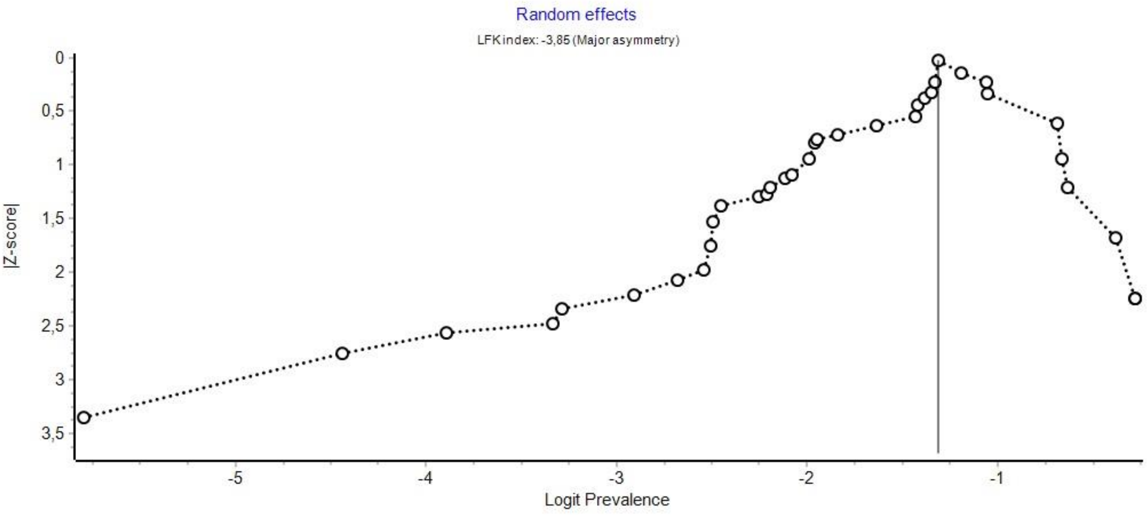

Supplementary figure 2 Doi plot with LFK index of feet-based evaluation
